# Common hepatic artery arises from superior mesenteric artery: bipode celiac trunk

**DOI:** 10.11604/pamj.2021.38.2.27425

**Published:** 2021-01-04

**Authors:** Danilo Coco, Silvana Leanza

**Affiliations:** 1 Department of General Surgery, Ospedali Riuniti Marche Nord, Pesaro, Italy,; 2 Department of General Surgery, Carlo Urbani Hospital, Jesi, Ancona, Italy

**Keywords:** Superior mesenteric artery, common hepatic artery, celiak bipode

## Image in medicine

Classic Common Hepatic Artery (CHA) from the celiac artery and the proper hepatic artery into right and left is seen in 55-60% of the population. A classification method was described by Michel *et al*. in 1955: I: standard anatomy ~60% (range 55-61%), II: replaced Left Hepatic Artery (LHA) ~7.5% (range 3-10%) III: replaced Right Hepatic Artery (RHA) ~10% (range 8-11 %) IV: replaced RHA and LHA ~1%,V: accessory LHA from Left Gastric Artery(LGA) ~10% (range 8-11%),VI: accessory RHA from Superior Mesenteric Artery (SMA) ~5% (range 1.5-7%),VII: accessory RHA and LHA ~1%,VIII: accessory RHA and LHA and replaced LHA or RHA ~2.5%, IX: CHA replaced to SMA ~3% (range 2-4.5%), X: CHA replaced to LGA ~0.5%. We present a case report of an 82-year-old Caucasian man who suffered from left colic tumor with a CHA, LHA, RHA arising from SMA: IX Michel Classification. Angio-CT Abdominal Scan demonstrated absence of complete celiac trunk. Bipode and CHA, LHA, RHA arising from SMA. 3D image: SMA+CHA, celiak bipode.

**Figure 1:**
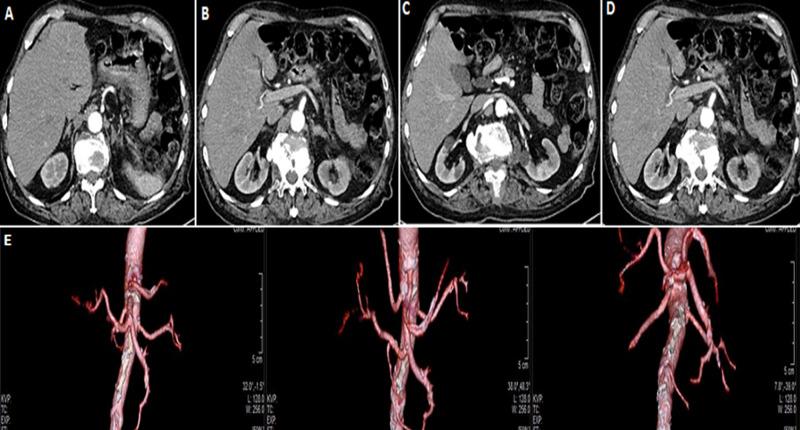
A) angio-CT abdominal Scan demonstrated absence of complete celiac trunk bipode; B) common hepatic artery (CHA); C) left hepatic artery (LHA); D) right hepatic artery (RHA) arising from superior mesenteric artery (SMA); E) 3D image: superior mesenteric artery (SMA) + common hepatic artery (CHA), celiak bipode

